# The Assessment of COVID-19 Pneumonia in Neonates: Observed by Lung Ultrasound Technique and Correlated with Biomarkers and Symptoms

**DOI:** 10.3390/jcm11123555

**Published:** 2022-06-20

**Authors:** Emil Robert Stoicescu, Diana Luminita Manolescu, Roxana Iacob, Simona Cerbu, Mirabela Dima, Emil Radu Iacob, Ioana Mihaiela Ciuca, Cristian Oancea, Daniela Iacob

**Affiliations:** 1 Department of Radiology and Medical Imaging, ‘Victor Babes’ University of Medicine and Pharmacy Timisoara, Eftimie Murgu Square No. 2, 300041 Timisoara, Romania; stoicescu.emil@umft.ro (E.R.S.); roxana.iacob@umft.ro (R.I.); cerbusimona@yahoo.com (S.C.); 2 Research Center for Pharmaco-Toxicological Evaluations, ‘Victor Babes’ University of Medicine and Pharmacy Timisoara, Eftimie Murgu Square No. 2, 300041 Timisoara, Romania; iacob.daniela@umft.ro; 3 Center for Research and Innovation in Precision Medicine of Respiratory Diseases (CRIPMRD), ‘Victor Babeș’ University of Medicine and Pharmacy, 300041 Timișoara, Romania; oancea@umft.ro; 4 Department of Neonatology, ‘Victor Babes’ University of Medicine and Pharmacy Timisoara, Eftimie Murgu Square No. 2, 300041 Timisoara, Romania; dima.mirabela@umft.ro; 5 Department of Pediatric Surgery, ‘Victor Babes’ University of Medicine and Pharmacy, Eftimie Murgu Square No. 2, 300041 Timisoara, Romania; radueiacob@umft.ro; 6 Pediatric Department, ‘Victor Babes’ University of Medicine and Pharmacy Timisoara, Eftimie Murgu Square No. 2, 300041 Timisoara, Romania; ciuca.ioana@umft.ro; 7 Department of Pulmonology, ‘Victor Babes’ University of Medicine and Pharmacy, Eftimie Murgu Square No. 2, 300041 Timisoara, Romania

**Keywords:** lung ultrasound, neonates, newborns, COVID-19, SARS-CoV-2, multisystem inflammatory syndrome

## Abstract

Newborns infected with SARS-CoV2 infection develop different symptoms in comparison with adults, but one thing is clear: some of the most common manifestations include cough and other respiratory symptoms that need to be evaluated. In these cases, lung ultrasound is a useful imaging technique that can evaluate the newborns’ lung damage caused by COVID-19 pneumonia and can be used for the surveillance of the patients as well, being non-irradiating and easy to use. Nineteen neonates who were confirmed as having SARS-CoV2 infection were investigated using this imaging tool, and the results were compared and correlated with their symptoms and biomarkers. The mean of LUSS was 12.21 ± 3.56 (S.D), while the 95% CI for the arithmetic mean was 10.49–13.93. The difference of an independent *t*-test between the LUSS for the patient who presented cough and the LUSS for the patient without cough was −4.48 with an associated *p*-value of *p* = 0.02. The Pearson’s correlation coefficient r = 0.89 (*p* = 0.03, 95% CI 0.0642 to 0.993) between the LUSS and IL-6 level showed a positive strong correlation. This reliable correlation between lung ultrasound score and inflammatory markers suggests that LUS could be used for monitoring inflammatory lung diseases in the future.

## 1. Introduction

SARS-CoV_2_ belongs to the Coronaviridae family, being the biggest monocatenary ARN virus discovered up until now. In the last two decades, coronaviruses were responsible for two pandemics—SARS and MERS [1]. The actual pandemic developed by the end of 2019 in Wuhan, China, where the first cases of COVID-19 pneumonia were confirmed. On 11 March 2020, WHO declared the infection with SARS-CoV2 a pandemic [2].

The data shows that the pediatric population was less affected when compared to adults, especially in developed countries, with a performing medical system [3]. However, the mortality of the pediatric population regarding viral infection was found to be high in underdeveloped countries [3]. Unlike adults, which can become infected by respiratory transmission, newborns can reach the infection through two types of transmission—vertical and postnatal [4]. 

The literature shows that vertical transmission is rare, while postnatal exposure to the virus gives a higher risk of infection, which can be nosocomial, intrafamilial or intracommunity [5]. The main diagnosis methods used on newborns are nasopharyngeal and rectal exudates, followed by RT-PCR tests on the probes. The incubation period of SARS-CoV2 infection is considered to be between 5–14 days; under this period false negative results could be found [5].

The clinical manifestations of COVID-19 in newborns are different than in the adult population, with gastrointestinal symptoms and a lack of appetite being the most common ones [5]. Other symptoms include fever, cough, and other respiratory symptoms, lethargy, diarrhea, vomiting, and rarely cardiovascular or cardiorespiratory shock [5]. The symptoms are milder than in adults, which accuse cough, dyspnea, and even respiratory insufficiency, accompanied by fever, myalgia, fatigue, and rarely digestive symptoms [6]. Preliminary data from Great Britain do not report any death in the neonatal population, as confirmed by other studies too [7,8]. In addition to the symptoms anteriorly described, the blood samples show leukocytosis, lymphopenia, thrombocytopenia, high CK values, especially CK-MB, and abnormal hepatic probes [5]. 

Complementary to the biologic samples, imagistic methods are very important techniques used in the diagnosis and surveillance of the newborn diagnosed with COVID-19 [9]. Existing data show that the preferred imaging technique used during the pandemic is computed tomography (CT) for the adult population. CT has the advantage of better visualization of the pulmonary parenchyma and the lesions caused by the viral infection [9,10].

Regarding newborns infected with SARS-CoV2, the imaging techniques used for evaluation include thoracic X-ray, CT, and lung ultrasound. Studies show that chest X-ray might be considered a first-intention tool for evaluating neonates, because of a lower radiation dose than CT [11,12]. Nevertheless, lung ultrasound has expanded in the past years [13], having many advantages when compared to X-ray and CT, such as:

- non-irradiating and non-invasive method;
- higher availability and accessibility, as well as lower costs;
- allows for multiple examinations, without the disadvantage of a cumulated radiation dose
- the existence of portable and ultraportable devices, which facilitates the examination of immobilized patients, especially in intensive care units [14,15].

During this pandemic, an increasing trend in using lung ultrasound for examining infected children and neonates has been observed, in concordance with the ALARA principles [16]. A few studies and systematic reviews present the advantages and main changes found by LUS in neonates and children with COVID-19 pneumonia, including:

- transverse physiologic A-lines—equivalent with unaffected parenchyma;
- erasing of A-lines;
- appearance of isolated vertical B-lines—interstitial edema;
- appearance of conflating vertical B-lines—alveolar edema;
- appearance of subpleural consolidations;
- diffuse pleural thickening and irregularities [17,18,19,20].

Other ultrasonographic changes, such as pleural effusion and pneumothorax have been reported only in a few cases [14,17,18].

Lung ultrasound score (LUSS) in neonatology is useful in predicting the need for surfactant therapy, the necessity of starting respiratory support, and the evolution of bronchopulmonary dysplasia [21].

This study aimed to find the most common changes detected with LUS on neonates with COVID-19 pneumonia and to correlate the findings with their symptoms and biomarkers, in order to prove the usefulness of this imagistic technique in neonates’ respiratory pathologies. 

## 2. Materials and Methods

The basis of this article consists of the analysis of the main symptoms and biological exploration of the newborns infected with SARS-CoV2, correlated with the imaging techniques. This prospective study was conducted at Neonatology and Neonatal Intensive Care Unit (NICU), ‘Pius Brinzeu’ Emergency County Hospital, between February 2020 and February 2022. The study was conducted in accordance with the Declaration of Helsinki and approved by the Ethics Committee of ‘Pius Brinzeu’ County Emergency Hospital (number 74/18 May 2020).

The inclusion criteria used for selecting the patients were:

- Newborns belonging to COVID-19 positive mothers that received a positive test at birth—vertical transmission;
- Neonates that developed SARS-CoV2 infection during their admission to the hospital—postnatal transmission;
- Newborns dismissed from the hospital who developed SARS-CoV2 infection in the first 28 days of life.
- The exclusion criteria were:
- Newborns with other respiratory pathologies;
- Neonates with SARS-CoV2 infection, associated with congenital respiratory diseases or cardiovascular malformations;
- Newborns that lacked lung ultrasound examinations, or those who had defective imaging techniques.

**Figure 1:**
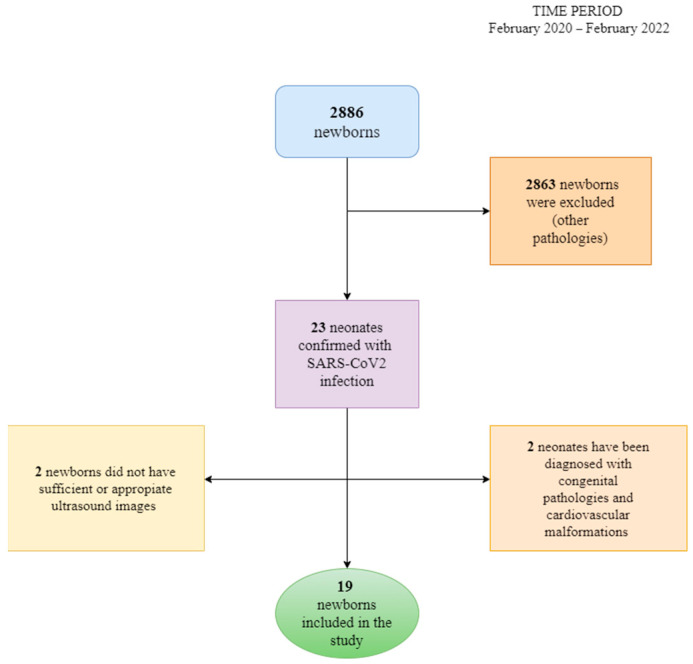
The algorithm of patients’ selection and exclusion criteria.

These criteria are presented in the scheme below (Figure 1).

The analyzed data were extracted from the hospital’s informatics program (InfoWorld), being registered in an Excel Microsoft Office table. The most important parameters that were taken into consideration for analysis were:

− gender;
− gestational age;
− anthropometric measurements (birth weight, cranial perimeter, thoracic perimeter);
− APGAR score;
− birth type—natural/C-section;
− pregnancy history and mother’s infection status;
− infection’s transmission type—vertical/postnatal;
− signs and symptoms of the infection in newborns (psychomotor agitation, sleepiness, fever, cough, rhinorrhea, weight loss, vomiting, diarrhea, loss of appetite, respiratory distress, oropharyngeal candidiasis);
− other associated pathologies;
− biological markers and inflammatory probes (hemoglobin, leukocytes, lymphocytes, thrombocytes, procalcitonin, C-reactive protein, CK, CK-MB, ferritin, LDH, hepatic transaminases, bilirubin, D-dimers, interleukin-6);
− bacterial and fungal cultures;
− imaging examinations;
− score of lung affection based on ultrasound.

**Figure 2:**
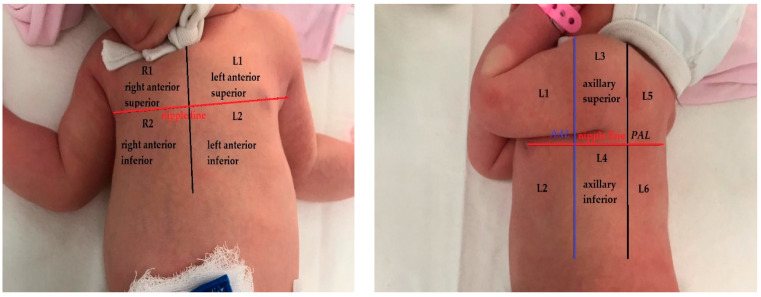

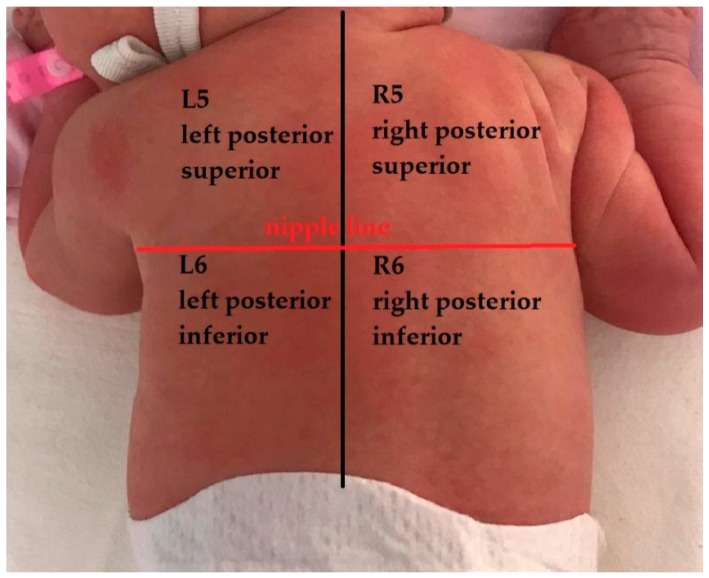
Lung areas—anatomical representation with 12 zones (AAL—anterior axillary line, PAL—posterior axillary line).

The lung ultrasound examination taken in the first days (2nd to 4th day) of infection was, in all the cases, conducted by a radiologist with two years of experience in lung ultrasound in newborns and children and supervised by a pediatric pulmonologist with nine years of experience in LUS. For understanding the lung changes in newborns with COVID-19 pneumonia when compared with normal lungs, literature data that approach this subject were researched. The examination was performed using a Samsung machine HM70 with a linear transducer (3–12 MHz) and a Samsung WS80 with a micro convex probe 6.0 MHz (4–12 MHz). An ultrasound machine soft for lung ultrasound was used with automatic enhancement performed with the same settings for every examined patient. A second lung ultrasound was performed for 9 neonates, but only the most representative one was used in the study, regarding symptomatology. The score described in this systematic review with a 12-area score and first described by Mongodi et al. [14,22] was used for semi-quantification of the lung lesions. The chest wall of newborns was divided into 12 areas with 6 areas for each hemithorax. There were two zones on the anterior chest wall (superior and inferior), two zones on the lateral (axillary zone) chest wall (superior and inferior), and two on the posterior chest wall (superior and inferior). A scheme of this representation is shown in the figure below—Figure 2. The nipple line was considered to be the demarcation line of the upper and lower regions. The score for each area ranged between 0–3 with exemplified images corresponding to each level—Table 1 below. Only one neonate was exanimated by CT.

All the data and analysis were processed using a licensed MedCalc version 20.026. The arithmetic mean and dispersion—standard deviation—were used as a central tendency indicator. The values are reported as mean ± standard deviation (S.D). The relationship between symptoms and LUS was documented using statistical tests, a Chi-squared (χ^2^) test with two variables (two-way classification) and crosstabs for a better illustration. The difference of sample means was evidenced by using the independent samples *t*-test. Also, the Pearson’s correlation coefficient r between the LUS and the principal markers of inflammation was calculated, with the following degree of correlation: if r is near ±1, the correlation is perfect; strong correlation with r between ±0.5 to ±1; medium correlation with r between ±0.30 and ±0.49; small correlation with r under ±0.29. The *p*-value < 0.05 was considered to be significant.

## 3. Results 

### 3.1. Demographic Data

From a total of 19 neonates with COVID-19 pneumonia admitted to the Neonatology and Intensive Care Unit (NICU) section of Clinical County Hospital ‘Pius Brinzeu’ in Timisoara, 12 neonates (63.17%) were of the male gender. From the point of view of their gestational age, only two neonates with SARS-CoV2 infection (10.52%) were preterm.

The mean weight of the neonates was 2936.84 ± 585.00, presented as the arithmetic mean ± standard deviation (SD), and 95% confidence intervals (CI) for the arithmetic mean were 2654.87 to 3218.80. One of the preterms weighted 950 g at a 27-week gestational age, a male twin, while the second one was a 31-week gestational age girl with a weight of 2140 g at birth. More detailed characteristics of the included patients can be found in Table 2 below.

Regarding the type of birth, 68.42% (13 neonates) were born by Cesarean section. Additionally, the mothers’ infection status was confirmed in 73.68% of cases, while for the rest of the neonates, their infections must be taken into discussion as community-acquired infection or a nosocomial one. Regarding the pregnancy’s history, two mothers presented with pathologies that can influence the development of fetuses, such as pregnancy arterial hypertension and hypothyroidism. 

Of the mothers with confirmed SARS-CoV2 infection (73.68% of cases), only three (15.78%) from all neonates included) were documented as having vertical transmission to the neonates. For the rest of the neonates (84.21%), the transmission was considered a postnatal one. The mean age (days) of the infected neonates was 13.25 ± 8.13 (95% C.I. 8.91–17.58).

### 3.2. Clinical and Biological Markers of COVID-19 Infection in Neonates

The most relevant neonates’ symptoms analyzed are presented in Table 3. Also, Table 4 illustrates the biomarkers and paraclinical data of neonates with COVID-19 pneumonia presented as mean ± S.D and 95% CI for the arithmetic mean.

Eight neonates (42.10%) were suspected of having a bacterial superinfection, so these had procalcitonin levels taken from their serum. The range of procalcitonin levels varied between 0.08 and ≥10 ng/mL (semiquantitative determination). The case with the highest procalcitonin level was further investigated by collecting a nasopharyngeal exudate, with a negative result on bacterial and fungal cultures. Furthermore, the nine neonates (47.36%) were also examined by collecting a nasopharyngeal exudate for a thorough examination. The results of the cultures were positive in only two cases (22.22%) with the involvement of Staphylococcus aureus and group D Streptococcus.

An elevated CK-MB level was found in three cases (15.78%), but with the results of the complementary cardiovascular examination being within normal limits. 

Another biological parameter taken into consideration for defining the inflammatory status was the level of interleukine-6 (IL-6), which in one case (5.26%) was increased over the cut-off point of 35 pg/mL. Also, D-dimer levels were raised in only one case (5.26%) with a value of 790 ng/mL.

One preterm was intubated at birth because of his status (950 g at birth, length 35 cm and a 6—APGAR score). The SARS-CoV2 infection was confirmed on the 10th day after birth. This preterm had developed respiratory distress syndrome, a severe form with a need of surfactant administration and mechanical ventilation. After three days, the ventilation mode was changed into nasal CPAP with FiO_2_ = 30%. Only three neonates needed supplementary O_2_ administration.

### 3.3. Lung Ultrasound Investigation, Score, and Correlation

All subjects included in the study were examined using the lung ultrasound technique, although only three neonates had radiography for an eventual comparison. Two of the radiographs were normal, but in one case it was described as a peribronchovascular accentuation of the bilateral pulmonary interstitium and adjacent minimal alveolar infiltrates. One neonate was examined by thoracic CT and revealed bilateral consolidations in the posterior segments

The lung ultrasound was performed on the first days of their admission to the Neonatology and Intensive Care Unit section and when the infection was confirmed by the positive result of a PCR test. For some patients (*n* = 9), another thoracic ultrasound in a varied period of time was performed, but for the results and statistics of this study, the relevant ones were integrated.

**Figure 3:**
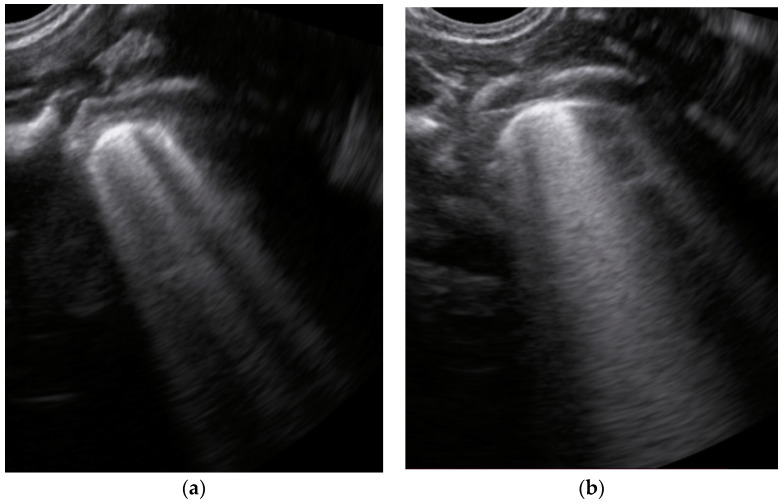
The lung ultrasound showed (**a**) sparse B-lines with small zones of pleural irregularities corresponding to a LUSS = 1; (**b**) confluent B-lines with aspect of ‘white-lung ‘corresponding to LUSS = 2.

**Figure 4:**
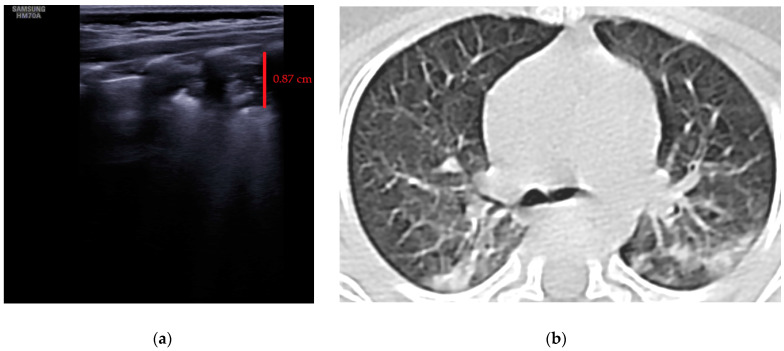
(**a**) The lung ultrasound showed a small consolidation area with the length <1 cm corresponding to a LUSS = 2; (**b**) The CT exam revealed bilateral consolidations in the posterior segments.

The LUS score (LUSS) varied between 4 and 18 points from a maximum of 36 points. The mean was 12.21 ± 3.56 (S.D), while the 95% CI for the arithmetic mean was 10.49–13.93. The changes described at lung ultrasound that appeared in a minimum of one area/neonate were:

- Erasing of A-lines—with a prevalence of 100%;
- Sparse B-lines (Figure 3a)—100%;
- Confluent or coalescent B-lines (Figure 3b)—57.89%;
- ‘White-lung’ (Figure 3b)—36.84%;
- Pleural abnormalities (irregularities, thickening, fragmented)—68.42%;
- Subpleural consolidation <1 cm (Figure 4a,b)—31.57%;
- Pleural effusion—5.26%.
- No large consolidation in the subjects included was reported.

**Figure 5:**
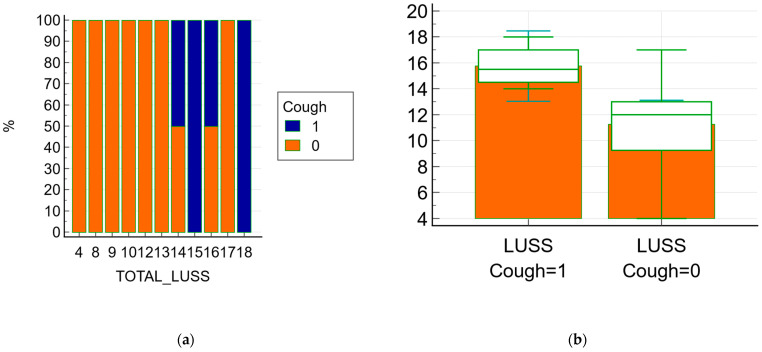
The relationship between symptoms (cough) and LUSS; 1 means present/affirmative and 0 means absence/negative (**a**) the frequencies chart of the Chi-squared test with a graph with a 100% stacked column; (**b**) Box-and-whisker of data comparison between LUSS at neonates with cough and LUSS at neonates without.

The relationship between symptoms and LUSS was documented using statistical tests, the Chi-squared (χ^2^) test in this case. The Chi-squared test for the trend between cough (present/absent) and LUSS has revealed that the χ^2^ trend was 6.083, DF = 1 with *p* = 0.01 (Figure 5a), while the Chi-squared test for the trend between respiratory distress syndrome (present/absent) and LUSS has revealed that the χ^2^ trend was 5.016, DF = 1 with *p* = 0.02. The relationship between psychomotor agitation and LUSS showed that the χ^2^ test was 16.851, DF = 10 with *p* = 0.07.

The difference of an independent *t*-test between the LUSS at the patient who presented cough and LUSS at the patient without cough was −4.48, with a 95% confidence interval from −8.18 to −0.77; the *t*-test statistic was 2.55, with 17 degrees of freedom and an associated *p*-value *p* = 0.02—Figure 5b. 

The difference between the sample means of LUSS in the patient who presented respiratory distress syndrome and LUSS in the patient without respiratory distress syndrome was −4.50, with a 95% confidence interval from −8.79 to −0.20; the *t*-test statistic was 2.21, with 17 degrees of freedom and an associated *p*-value of *p* = 0.04. Moreover, the difference between the sample mean of LUSS and non-physiological weight loss was reported as −3.05, 95% CI −6.39 to 0.27; *t* = −1.936; DF = 17, *p* = 0.06.

**Figure 6:**
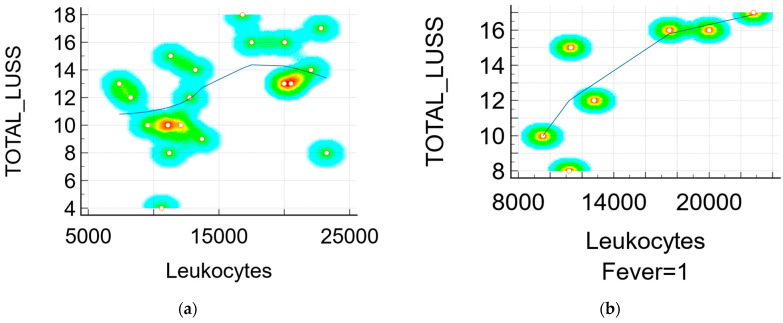

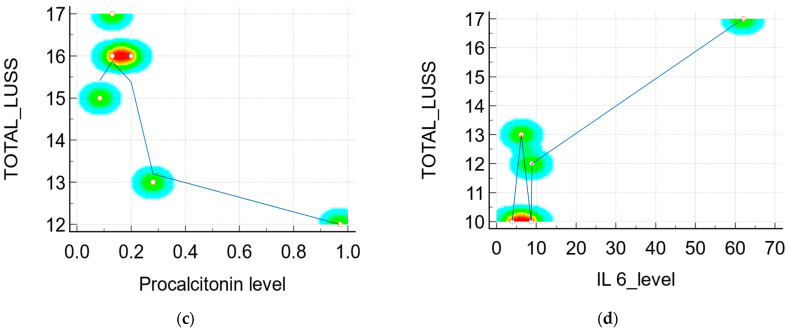
Scatter diagram with heat map of correlation between LUSS and biomarkers: (**a**) LUSS and number of leukocytes from all neonates; (**b**) LUSS and number of leukocytes from the neonates with symptoms—a positive linear correlation; (**c**) LUSS and procalcitonin level—a negative linear correlation; (**d**) LUSS and IL-6 level—a positive linear correlation.

**Figure 7:**
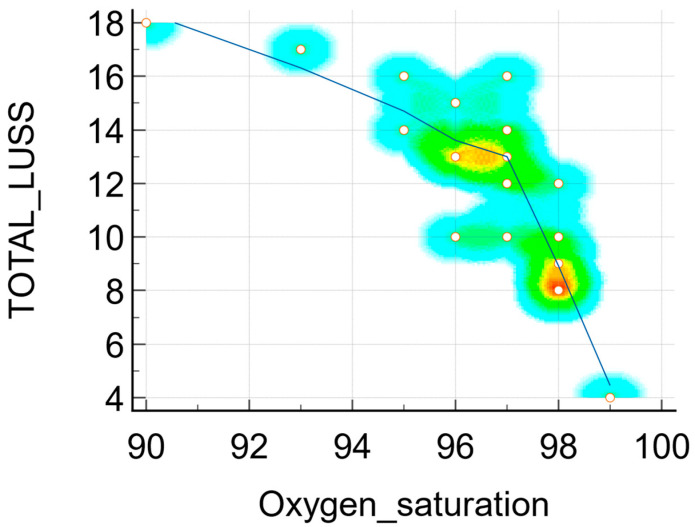
Scatter diagram with a heat map of correlation between LUSS and O_2_ saturation.

The Pearson’s correlation coefficient r between the LUSS and the principal markers of inflammations were:

- r = 0.38 (*p* = 0.10, 95% CI 0.0834 to 0.714) between the LUSS and number of leukocytes—Figure 6a;
- r = 0.79 (*p* = 0.03, 95% CI 0.105 to 0.968) between the LUSS and number of leukocytes at symptomatic neonates (with fever)—Figure 6b;
- r = 0.36 (*p* = 0.12, 95% CI −0.102 to 0.704) between the LUSS and CK level;
- r = 0.92 (*p* = 0.07, 95% CI −0.306 to 0.998) between the LUSS and CK level at symptomatic neonates;
- r = 0.33 (*p* = 0.21, 95% CI −0.211 to 0.724) between the LUSS and CRP level;
- r = 0.16 (*p* = 0.50, 95% CI −0.325 to 0.587) between the LUSS and LDH level;
- r = −0.79 (*p* = 0.06, 95% CI −0.976 to 0.0531) between the LUSS and procalcitonin level (Figure 6c), but in this case, the statistic was done with only six symptomatic neonates who had their procalcitonin level determined by a quantitative measurement;
- r = 0.89 (*p* = 0.03, 95% CI 0.0642 to 0.993) between the LUSS and IL-6 level (Figure 6d), but in this case the statistic was done with only five symptomatic neonates (with fever, cough, and rhinorrhea) who had their IL-6 level determined;
- r = −0.77 (*p* = 0.0001, 95% CI −0.907 to −0.489) between the LUSS and O_2_ saturation level—Figure 7.

## 4. Discussion

The neonates included in the study developed an asymptomatic, mild, or moderate form, without the need for orotracheal intubation due to SARS-CoV_2_ infection. In accordance with Musolino et al. and Smargiassi et al., the importance of LUS is demonstrated in differentiating mild and moderate forms of COVID-19 pneumonia in children, due to the fact that children with mild forms mostly have a normal LUS pattern with no pathological findings [23,24]. The severity of the admitted cases has been reduced when compared to other similar studies that describe the need for intubation and even death among newborns with COVID-19 pneumonia [25]. This fact is also confirmed by our study results: a higher mean birth weight (2936.84 g vs. 2394 g), the percentage of preterm births was lower (10.52% vs. 41%), the mean of APGAR score was higher (8.71 vs. 8), and there was only one preterm (5.26%) who needed respiratory support at birth, compared to a 25% rate of intubation at birth in the other study [25]. 

Most of the frequent transmission types were postnatal ones, according to the idea that vertical transmission was at low risk [5]. Moreover, the rate of postnatal transmission reported by Ibarra-Ríos et al. was 91%, pretty much similar to the 84.21% found in our report [25].

The inflammatory status was well defined by the leukocytes, lymphocytes, and the level of the following biomarkers CK, CRP, LDH, AST, ferritin, and IL-6. For example, the mean of leukocytes (14,807.77/µL) was higher than the others reported (11,900/µL), but with differences in the mean of lymphocytes (6207.77/µL vs. 4032/µL) and neutrophils (6748.88/µL vs. 5936/µL) [25]. This difference can be explained by the changes in the immune system during the SARS-CoV2 infection, with the possibility of associated bacterial infection (and increase of the neutrophil level). Moreover, 12 neonates (63.15%) received antibiotic treatment. Additionally, the Pearson’s correlation coefficient r between LUSS and procalcitonin (r = −0.79; *p* = 0.06) for symptomatic newborns with determining levels revealed a large negative relationship, but with low–moderate strength evidence. This fact can be explained by an additional bacterial non-respiratory infection developed along with the SARS-CoV2 infection. In this case, procalcitonin level and neutrophils suggest that neonates developed a bacterial infection without lung expression. Moreover, the lack of large consolidation on LUS that was correlated with bacterial cross-contamination confirm the idea of an additional non-respiratory infection during SARS-CoV2 infection in newborns [26]. Neither small (<1 cm), nor large consolidations were described in the newborns and pediatric patients with COVID-19 pneumonia [14,26]. According to Buonsenso et al., the changes in lung injuries (consolidations, air bronchogram) in community acquired pneumonia was correlated with treatment response more than with the laboratory findings, which can explain the inflammatory status of newborns included [27].

Psychomotor agitation was the most frequent symptom developed by neonates with SARS-CoV2 infection, and there was a possible relationship found between this symptom and LUSS (χ^2^ test was 16.851, DF = 10 with *p* = 0.07). With a *p*-value between 0.05 and 0.1, there is evidence against the null hypothesis (the two factors are independent), in favor of a relation of dependence.

The respiratory symptoms such as cough had an equivalent impact in terms of lung damage imaging, according to the Student’s *t*-test test results (the difference was −4.48 with an associated *p* = 0.02). So, the patients with symptoms (cough) had a mean average (M.A.) of LUSS (15.75) higher than the mean average of LUSS in neonates without symptoms (11.26), which was statistically significant. Similar to this fact, the neonates who presented with respiratory distress syndrome had an increased LUSS score than the neonates without (M.A. 16.00 vs. 11.50, *p* = 0.04). The three neonates who needed supplementary oxygen administration had a high score (18, 16 and 17); in these cases a cut-off limit of LUSS of more than 15 could predict the necessity of additional oxygen administration. Moreover, there was a strong negative correlation (r = −0.77, *p* = 0.0001) between LUSS and O_2_ saturation levels, which underlines the idea of lung ultrasound being a non-invasive surveillance method for neonates with respiratory pathology.

An interesting connection was found between the neonates with SARS-CoV_2_ infection who presented with non-physiological weight loss and who had an increased LUSS (M.A. 14.14 vs. 11.08, *p* = 0.06). Probably, the infection influenced the normal development of the neonates, causing a temporary slowdown in weight gain. A higher number of enrolled subjects could give more strength to statistical results and define a direct relationship.

The elevated CK and CK-MB levels in neonates with COVID-19 pneumonia were also reported in other three studies, outlining the idea that newborns develop myocardial dysfunction during SARS-CoV2 infection [14,18,23]. This phenomenon along, with the elevated values of the inflammatory status, especially procalcitonin levels for bacterial infection, advocates for the definition of the multisystem inflammatory syndrome in SARS-CoV2 infection in neonates.

The inflammatory status (leukocytes’ number) for all neonates (symptomatic or not) could be correlated with LUSS (r = 0.38, *p* = 0.10) to a moderate degree and with low statistical strength. However, this case should be taken in account with the bacterial infection which could evolve simultaneously with COVID-19 pneumonia. For the symptomatic neonates, there was a high correlation between leukocytes and LUSS (r = 0.79, *p* =0.03) between the LUSS and the number of leukocytes in symptomatic neonates), pretty much similar to the correlation between LUSS and CK levels in neonates with cough.

The elevated level of IL-6 was strongly correlated with LUSS (r = 0.89, *p* = 0.03) with moderate statistical strength, turning it into an almost accurate marker of the correlation of lung damage with its level. The changes described by lung ultrasound in neonates with COVID-19 pneumonia were summarized in a systematic review [14]. When compared with the review’s results, from the table below (Table 5), the present study’s results fit in their range percentage of changes.

Furthermore, LUSS was an additional element taken into account for the management of SARS-CoV2 infection in newborns, adjusting the therapy and intervention, and also reducing the dose of radiation for these newborns (only three X-rays and one CT-exam were performed). 

For nine neonates, we performed another lung ultrasound examination during the hospitalization, with a lower LUSS in all cases, which outlined the idea of the rapid ultrasonographic healing of newborns [28].

The present study’s mean of LUSS was 12.21 ± 3.56 (S.D) which is higher than Li et al.’s study (8.40 ± 1.70) and was included in the range of Ibarra-Ríos et al. study (11.2–16) which is scored using a 10-area score [18,25]. The most common signs found in the newborns included in study were represented by B-lines changes, a fact confirmed also by Musolino et al. [24,29]. Moreover, unlike adults, pleural consolidations and pleural effusion are rare in in the infected children, seen also in our results [24,29].

### 4.1. Limitation of Study/Weakness

One of the limitations of the study was the relatively small sample size with only 19 neonates with COVID-19 pneumonia. A large number of subjects and a homogeneity of data would have led to results with a stronger statistical power. Also, a follow-up study of neonates and their lung ultrasound changes would have led to a better correlation between inflammatory status and ultrasound severity score (LUSS).

### 4.2. Further Directions 

Even though this is one of the largest groups of newborns infected with SARS-CoV2 infection that has been analyzed also from an ultrasound point of view (the largest from Eastern Europe), there are many future directions that can be insisted on, such as finding biomarkers with a higher sensitivity and specificity that are correlated with pulmonary ultrasonographic changes in various respiratory pathologies. Moreover, the greater the evidence of the effectiveness of the use of lung ultrasound in the analysis and follow-up of patients with respiratory pathology, especially newborns, infants, and children, the more popular it will become.

## 5. Conclusions

The reliable correlation between lung ultrasound score and highly sensitive inflammatory markers, such as the IL-6 level and leukocytes, could suggest the further use of LUSS in monitoring inflammatory lung diseases. With current advances, in the near future, lung ultrasound could be used as a non-invasive surveillance method in neonates and children affected by pneumonia.

## Figures and Tables

**Table 1:** LUS score, corresponding image, and description of image.

LUS Score	0	1	2	3
Corresponding image	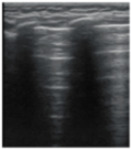 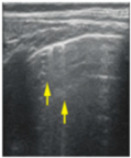	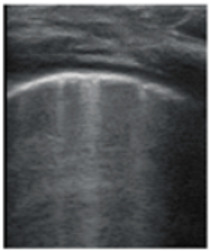 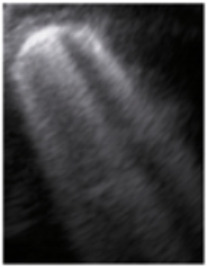	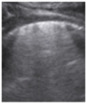 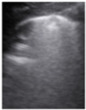	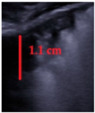 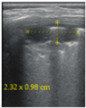
Description of images	Physiological A-lines (upper image) One or two B-lines per intercostal space (marked with yellow arrows) with smooth pleural line (lower image)	More than two B-lines per intercostal space with irregular or thickened pleura	Coalescent/confluent B-lines (upper image)/‘white lung’ or small subpleural consolidations (<1 cm—lower image)	Large consolidations (>1 cm) ± air bronchogram—the image is from a newborn not include in the study with bacterial pneumonia

**Table 2:** The baseline characteristics of infected neonates presented as mean ± S.D and 95% CI for the arithmetic mean.

Neonates’ Characteristics	Mean ± S.D.	95% CI for the Arithmetic Mean
Age (days) when infection was confirmed	11.15 ± 8.93	6.85 to 15.46
Weight at birth (g)	2936.84 ± 585.00	2654.87 to 3218.80
Length at birth (cm)	49.50 ± 5.24	46.16 to 52.83
Head circumference at birth (cm)	33.31 ± 3.36	31.05 to 35.57
Thoracic circumference at birth (cm)	31.81 ± 3.62	29.38 to 34.25
APGAR score in the 1st minute	8.71 ± 0.99	8.14 to 9.28
Positive PCR tests	2.78 ± 1.51	2.06 to 3.51
Days of hospitalization	11.73 ± 7.26	8.23 to 15.23

**Table 3:** The symptoms and comorbidities of neonates with COVID-19 pneumonia presented as the number of patients and percentage (%) of the lot.

Neonates’ Symptoms	*n* = 19 (Percentage %)
Psychomotor agitation	12 (63.15)
Excessive sleepiness/lethargy	5 (26.31)
Fever (≥37.5 °C)	7 (36.84)
Cough	4 (21.05)
Rhinorrhea	9 (47.36)
Non-physiological weight loss	3 (15.78)
Episodes of diarrhea	4 (21.05)
Vomiting	2 (10.52)
Loss of appetite	10 (52.63)
**Associate pathologies and comorbidities**	
Respiratory distress syndrome	3 (15.78)
Oral candidiasis	9 (47.36)
Conjunctivitis and dacryocystitis	4 (21.05)
Cryptorchidism	1 (5.26)
Congenital hydrocephalus	1 (5.26)
Transient tachypnea of the newborn	1 (5.26)
Retinopathy	1 (5.26)

**Table 4:** The biomarkers and paraclinical data of neonates with COVID-19 pneumonia presented as mean ± S.D and 95% CI for the arithmetic mean.

Biomarker (Unit Measurement)	Mean ± S.D.	95% CI for the Arithmetic Mean
Hemoglobin (g/dL)	14.22 ± 3.00	12.72 to 15.71
Leukocytes (×10^9^/L)	14,807.77 ± 5294.01	12,175.12 to 17,440.43
Lymphocytes (×10^9^/L)	6207.77 ± 2492.12	4968.47 to 7447.08
Neutrophiles (×10^9^/L)	6748.88 ± 5018.63	4253.18 to 9244.59
Thrombocytes (×10^9^/L)	314,944.44 ± 151,973.09	239,370.00 to 390,518.88
CK (U/L)	344.50 ± 446.97	122.22 to 566.77
LDH (U/L)	546.17 ± 131.83	478.39 to 613.95
AST (U/L)	75.88 ± 62.33	44.88 to 106.88

CK = Creatine kinase; LDH = Lactate dehydrogenase; AST = Aspartate aminotransferase; ALT = Alanine aminotransferase; CRP = C-reactive protein.

**Table 5:** The comparison between systematic review results and present study results.

Lung Ultrasound Change	Systematic Review’s Results [14]	Present Study’s Results
Erasing of A-lines	62.8–100%	100%
Sparse B-lines	55.3–100%	100%
Confluent or coalescent B-lines	1.5–66.6%	57.89%
Pleural abnormalities (irregularities, thickening, fragmented))	21.9–100%	68.42%
Subpleural consolidation <1 cm	1.5–66.6%	31.57%
Pleural effusion	Not reported	5.26%

## Data Availability

The data are contained within the article. Additional information is available on request from the first author. The data are not publicly available due to patient privacy requirements of clinical data.
